# Ethel Parsons’ biographical characteristics: leadership in American and Brazilian nursing

**DOI:** 10.1590/1980-220X-REEUSP-2022-0320en

**Published:** 2023-01-06

**Authors:** Angela Aparecida Peters, Maria Angélica de Almeida Peres, Maria Itayra Padilha, Patricia D’Antonio, Pacita Geovana Gama de Sousa Aperibense, Tânia Cristina Franco Santos, María Sagrario Gómez-Cantarino

**Affiliations:** 1 Universidade Federal do Rio de Janeiro, Escola de Enfermagem Anna Nery, Programa de Pós-Graduação em Enfermagem, Rio de Janeiro, RJ, Brazil.; 2 Universidade Federal Santa Catarina, Programa de Pós-Graduação em Enfermagem, Florianópolis, SC, Brazil.; 3 University of Pennsylvania, School of Nursing, Family and Community Health Department, Philadelphia, PA, United States of America.; 4 Universidade Federal do Rio de Janeiro, Centro Multidisciplinar UFRJ-Macaé, Instituto de Enfermagem, Macaé, RJ, Brazil.; 5 Universidad de Castilla-La Mancha, Departamento de Enfermería, Fisioterapia e Terapia Ocupacional, Toledo, Castilla-La Mancha, Spain.

**Keywords:** Biograph, Schools, Nursin, History of Nursin, Public Healt, Biografía, Facultades de Enfermería, Historia de la Enfermería, Salud Pública, Biografia, Escolas de Enfermagem, História da Enfermagem, Saúde Pública

## Abstract

**Objective:**

to reconstruct Ethel Parsons’ biographical aspects in the centenary of the technical cooperation mission for nursing development in Brazil.

**Method:**

biographical research, carried out using the historical sources analysis method, which consists of reading and interpreting the collected documents.

**Results:**

from a renowned family, Ethel Parsons was head of public health services and worked for the American Red Cross before being appointed to coordinate the Rockefeller Foundation mission in Brazil, where she inaugurated the Anglo-American model of nursing. For ten years, Parsons dedicated herself to leading such a mission, which resulted in implementation and dissemination, by decree, of the Anglo-American model of nursing. In 1931, she returned to her country, where she died in 1953.

**Conclusion:**

Ethel Parsons stood out in the 20th century as a woman and a nurse, leading public health care services in the USA and Brazil. Her biography demonstrates an ideal of professionalization and science to be conquered by nursing in the care and educational scenario, which influenced the design of a collective identity for Brazilian nursing.

## RESUMO

**Objetivo:**

reconstruir aspectos biográficos de Ethel Parsons no centenário da missão de cooperação técnica para o desenvolvimento da enfermagem no Brasil.

**Método:**

pesquisa biográfica, realizada pelo método de análise de fontes históricas, que consiste em leitura e interpretação dos documentos coletados.

**Resultados:**

de família renomada, Ethel Parsons ocupou chefias de serviços de saúde pública e atuou na Cruz Vermelha Americana antes de ser indicada para coordenar a missão da Fundação Rockefeller no Brasil, onde inaugurou o modelo anglo-americano de enfermagem. Por dez anos, Parsons se dedicou a liderar tal missão, que resultou na implantação e difusão, por força de decreto, do modelo anglo-americano de enfermagem. Em 1931, retornou a seu país, onde faleceu em 1953.

**Conclusão:**

Ethel Parsons se destacou no século XX como mulher e enfermeira, liderando serviços assistenciais de saúde pública nos EUA e no Brasil. Sua biografia demonstra um ideal de profissionalização e ciência a ser conquistado pela enfermagem no cenário assistencial e educacional, que influenciou no delineamento de uma identidade coletiva da enfermagem brasileira.

## RESUMEN

**Objetivo:**

reconstruir aspectos biográficos de Ethel Parsons en el centenario de la misión de cooperación técnica para el desarrollo de la enfermería en Brasil.

**Método:**

investigación biográfica, realizada mediante el método de análisis de fuentes históricas, que consiste en la lectura e interpretación de los documentos recopilados.

**Resultados:**

de una familia renombrada, Ethel Parsons fue jefa de servicios de salud pública y trabajó para la Cruz Roja Americana antes de ser designada para coordinar la misión de la Fundación Rockefeller en Brasil, donde inauguró el modelo angloamericano de enfermería. Durante diez años, Parsons se dedicó a liderar tal misión, que resultó en la implementación y difusión, por decreto, del modelo angloamericano de enfermería. En 1931 regresó a su país, donde falleció en 1953.

**Conclusión:**

Ethel Parsons se destacó en el siglo XX como mujer y enfermera, al frente de los servicios públicos de salud en EUA y Brasil. Su biografía demuestra un ideal de profesionalización y ciencia a ser conquistado por la enfermería en el escenario asistencial y educativo, lo que influyó en el diseño de una identidad colectiva para la enfermería brasileña.

## INTRODUCTION

The need to build Ethel Parsons’ biographical narrative is evidenced, mainly, by her significant role in the Brazilian National Department of Public Health (DNSP – *Departamento Nacional de Saúde Pública*), alongside sanitary physician Carlos Chagas, in technical cooperation that developed modern nursing in Brazil. Little is known about this professional, who was the first female nurse to hold a public position of national leadership, such as the DNSP Nursing Service, and mentor of a training model for nurses that became the desired standard of teaching for nursing in Brazil.

Even with Brazilian nursing advancing in nursing history, especially in the last 40 years, Ethel Parsons’ biography was not sufficiently known to reveal characteristics of her social and professional identity. A large part of historical records and reports about Ethel Parsons’ life expose moments of her professional activity over the 10 years she spent in Brazil (1921–1931).

The Technical Cooperation Mission for the Teaching of Nursing in Brazil, or “Parsons Mission”, was defined by many researchers as the milestone of modern nursing implementation in the country, which is corroborated by the authors of this study, supported by the fact that the said mission, when opened the DNSP nursing school in 1923, established the minimum curriculum and the necessary conditions to become a nurse, both prepared by the National League of Nursing Education and published in the Goldmark Report (1923)^(1,2)^.

Moreover, the DNSP nursing school, currently *Escola de Enfermagem Anna Nery* (Anna Nery Nursing School), *Universidade Federal do Rio de Janeiro* (UFRJ), was recognized as an “official standard school” by Decree 20,109 of June 15, 1931 of the Federal Government, making it became a model for existing and future Brazilian nursing schools, remaining in this condition until 1949, when Law 775 was enacted, which established criteria for undergraduate and nursing assistant courses. Despite this, several schools created later continued with the same nursing teaching model^(3,4)^.

When dealing with modern nursing development in Brazil, it is worth mentioning the opening of a course inspired by the Nightingale model in the city of São Paulo in the 19^th^ century, linked to *Hospital Samaritano*. Studies will probably still be produced on the reasons that prevented disseminating the aforementioned model in Brazilian society at that time. It is known that Parsons Mission, in the 20^th^ century, was a proposal by the Federal Government to carry out a Health Reform and, for this reason, facilitated in its purposes of creating, disseminating and implementing a nursing model in society, with special emphasis on public health nursing (PH), which is fundamental for constructing professional nursing practice by the precursor of modern nursing^(5)^. In fact, nursing education initiatives prior to Parsons Mission are recorded in Brazilian nursing historiography, which leads us to reaffirm a reflection carried out by one of the authors of this study on the imprisonment of advances in nursing knowledge to the Nightingale model, when, in the USA, even before the arrival of this nurse’s ideas, there was already a nursing with high quality standard^(6)^.

The Parsons Mission committed itself to implementing the most modern in the world in terms of nursing as a female profession. As far as possible, given Brazil’s conservative ideals in the 1920s, Ethel Parsons, through nursing school, trained professionals to qualify, in addition to assistance in PH, assistance in general and specialized hospital environments. This qualification gave visibility to graduated nurses’ identity in the health field, which received formal recognition in 1937, when their training began to take place in the university space, with the insertion of the referred school at *Universidade do Brasil* (University of Brazil), currently UFRJ^(7)^.

In a philosophical view on professional identity in nursing, a study underlines Nightingale’s modernity in the professional profile of Brazilian nursing and the concepts arising from Ethel Parsons’ propositions that currently permeate professional knowledge/doing, representing epistemological bases that remain, despite the social crises experienced in the country, which affected the historical-evolutionary advances of nursing^(8)^.

The discovery of characteristics of Ethel Parsons’ personal trajectory, from the construction of a doctoral thesis entitled “*Missão Parsons: da implantação de um modelo de enfermeira de SP* às *bases da profissionalização da Enfermagem brasileira* (1921–1931)”, contributed to rereading documents, making it possible to know more influences of this leader of Brazilian nursing, allowing to assess identity elements of a nursing model implemented in Brazil. Even if some biographical elements are subjective, Ethel Parsons personality’s value for nursing by itself would justify this study on her biography.

Considering the above, the question by the authors emerged: what sociocultural characteristics served as the basis for building Ethel Parsons’ professional identity, a nurse appointed to develop Brazilian nursing? This study aimed to reconstruct Ethel Parsons’ biographical aspects in the centenary of the Technical Cooperation Mission for the Teaching of Nursing in Brazil. Knowledge gaps on the subject range from different interpretations for her paternal surname to lack of information about her complete professional training, going through her social origins and her image, since, in a simple internet search, there are photographs of homonymous people identified as her, which has been corroborating with historical error dissemination in academic works.

## METHODS

### Study Design

This is biographical research, carried out using historical sources analysis, which consists of reading and interpreting the documents collected^.^ It should be clarified that the field of knowledge of biographical research seeks to reaffirm the specificity and centrality of a biographical fact in individuation and socialization processes. Social conditioning, suffered by every human being, are important factors in defining life stories^(9)^. Biographies contribute to the debate about the profession in professional identity construction of nursing, as they allow highlighting the legacy left by characters who influenced and continue to influence care practices, research and education in nursing^(10)^.

### Documentary Source Collection

**Chart 1.**
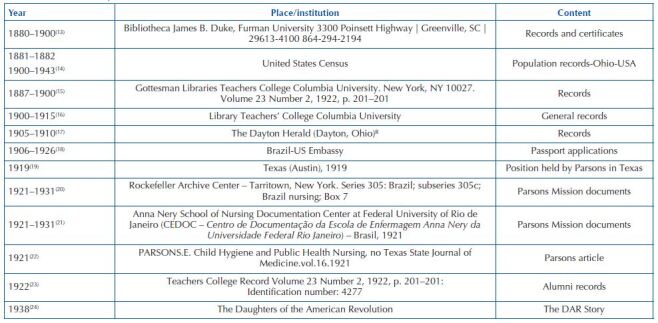
Documentary sources about Ethel Parsons’ life – Juiz de Fora, MG, Brazil, 2020/2021.

The data that support this research were collected in two countries, from July 2020 to August 2021. In Brazil, the documents were collected at the Anna Nery School of Nursing Documentation Center at UFRJ; in the United States (USA), the documents were found in the following institutions: US Embassy (American customs responsible for immigration and travel services); Rockefeller Archive Center – Tarritown, New York; Walworth Harrinson Public Library – Greenville; The Teachers College Record Archives; The Gottesman Libraries – Teachers College Columbia University; James B. Duke Library, Furman University, Greenville; Denison University – Granville. Chart 1 below presents the documentary sources used for producing this research.

Documentary corpus selection occurred from the study thematic and temporal cut and observation of principles of relevance, sufficiency, exhaustiveness, representativeness, homogeneity and organization by sectors in relation to the problem and the studied theme^(11)^. Incomplete documents that did not allow authorship or context identification in which they were produced were excluded. Reports, official records, journalistic sources published by local press and Ethel Parsons’ personal documents were included.

### Source Treatment and Analysis

After being selected, the documentary corpus was categorized and was subjected to a thorough and exhaustive reading, cataloged in a spreadsheet by chronology, type and subject. Duplicate sources were removed, and triangulation between sources was established, which led to convergent validity of the findings, ensuring greater reliability due to combination of methodological procedures^(11)^. It should be noted that international sources were separated in chronological order and translated into Brazilian Portuguese by a professional English-speaking translator.

The sources were analyzed through documental criticism (internal and external), aiming to assess their credibility and representativeness. At this stage, source veracity was verified, observing support and document nature, origin, originality, authenticity, and archive credibility^(12)^.

### Ethical Aspects

The documents mentioned in this article belong to archives of Brazilian and North American institutions that attest to source internal and external credibility and are open for consultation, according to their specific rules.

## RESULTS

The search resulted in a total of 12 documents, as shown in Chart 1, cited throughout this text.

### Character Ethyle Estelle Shaman Arises

**Figure 1.**
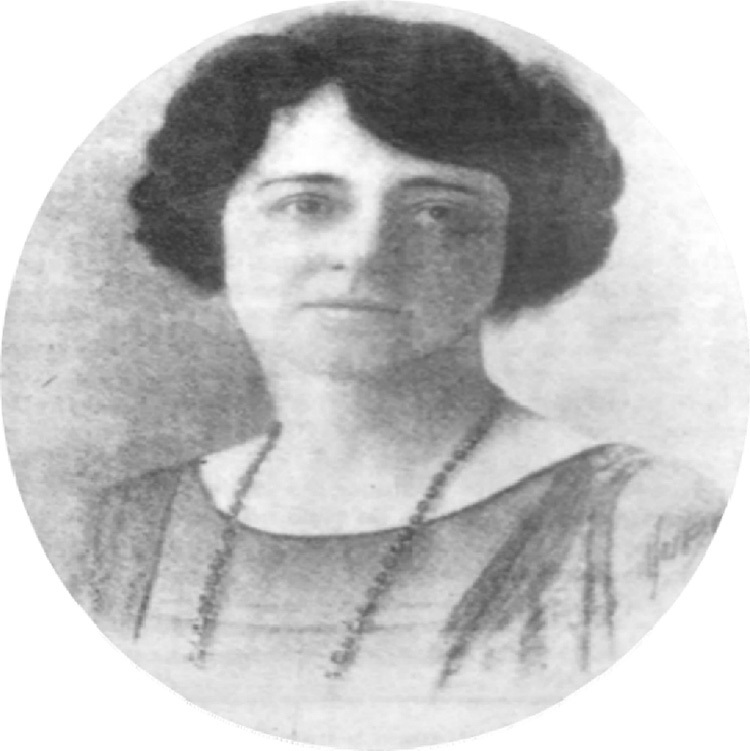
Ethel Parsons’ portrait donated to the Brazilian National Department of Public Health Nursing school.

Source: Anna Nery School of Nursing Documentation Center, UFRJ, RJ, Brazil, 1921. Photograph 7.03.1752.1.^21^

In order to trace nurse Ethel Parsons’ biography (Figure 1), an exhaustive search for documents both in Brazil and in the USA was necessary, her country of origin, in order to uncover possible nominal traces that would guarantee the intended redemption. Lack of information after search using her name, as spelled in the documents found in Brazil, led to the need for a thorough analysis of “cursive handwriting” on the record file of her personal history as Rockefeller Foundation employee, completed by Ethel Parsons herself, which contains her name and signature. This highlight is necessary to understand the facts that defined Ethel Parsons’ birth name, a key element to find the documents that make up this study.

Registered at birth as Ethyle Estelle Shaman, of Swiss descent, by her paternal grandparents, nurse Ethel Parsons was born on September 18, 1882 and died on October 13, 1953, aged 71. She was born in Benington Village, located south of Locke and north of Granville, Ohio, USA^(13)^.

With regard to her date of birth, it appears in a document filled out by her as the year 1884. However, official records of residents of Ohio show that, until 1881, only her parents lived in Ethel’s family home, and from 1882 onwards, her name also appeared at the same residential address, which indicates her birth in the period between the 1881/1882 censuses^(13,14)^.

Daughter of Emmanuel Elias Shaman and Carrie Arnold Shaman, her parents separated in 1900 when she was 18. Ethel remained living with her mother, who married James McCann for the second time in 1904, who became her stepfather^(13,14)^. Her mother is identified as “matron” and worked as a supervisor at the Orphanage Support Services Organization (OSSO), in Xenia/Ohio, the city where Ethel lived after separating from her first husband, having retired as a nurse^(14,17)^. Carrie was a descendant of the Daughters of the American Revolution (DAR) through her maternal grandfather, Jacob Arnold, an important character in the revolution who served as a combatant. Ethel, as a great-granddaughter, inherited the position of DAR, holding the title Patriots of America in the family. The importance of Ethel’s family in American history was evidenced in the main newspapers of the states of Ohio, Texas, New Mexico and California since her adolescence^(15,17,24)^.

Until she became a teenager, the name “Ethyl” was what appeared on forms and documents from the institutions Ethel attended. From 1897, when she was 18 years old, the media referred to her as Ethel (with the letter “e” instead of “y” in Ethyle)^(13–15)^. The same fact occurred in relation to her mother’s name, Carrie, which in some documents was written as Carried (with the addition of the letter “d” at the end). Likewise, we evidence inconsistencies in her father’s surname, Shaman, written in different ways in different documents, namely: Shenon, Shernon, Sheron, Sharon, Sharnon^(13,15,17)^.

In the Parsons Mission documents, already having her married surname (Parsons), she used to abbreviate her paternal surname (Shaman). This abbreviation caused the handwritten letter to be interpreted by Brazilian researchers sometimes as the letter “s” (Ethel S. Parsons)^(25)^ sometimes as the letter “o” (Ethel O. Parsons)^(26)^.

The identification of the first letter of Ethel Parsons’ paternal surname, despite seeming to be a relatively simple detail or of little relevance, was one of the greatest obstacles for the research to flow. In the Personal History Record Form, which she completed for the Rockefeller Foundation (RF), it was possible to observe that, at some later time, the letter “s” was added, also in cursive, with a pen of a different color from the original, the spelling “haman”, forming the surname Shaman^(20,21)^.

In a close look at the aforementioned registration form, Ethel Parsons declares herself a widow (widow), which confirms the surname Parsons as being her husband’s. As usual, in marriages at the time, women used the surname of their husbands, and this is confirmed in documents from Brazil (correspondence, letters, memorandums), she being referred to as “Mrs. Parsons”. The acronym “Mrs” is used to mention a married woman, unlike the acronym “Ms”, used to identify a single woman^(13,20,21)^.

From the discovery of Ethel’s paternal surname, new contacts were made with the education and professional training institutions mentioned in the same file, in which, in previous searches by the abbreviated and married name, no data on Ethel Parsons were found. Once knowing her paternal surname, it was possible to identify Ethel, before Ethyle, in one of the lists of students of the educational institution she attended^(15)^.

As completed in the RF form, Ethel Parsons attended from 1896 to 1899 at Granville High School, located in the city of Granville, Ohio. From 1899 to 1902, she studied at Granville Female College, a college for girls whose parents were part of or supported the Presbyterian doctrine, where a broad culture was acquired, including knowledge of Music, Arts and Languages and Literature; from 1902 to 1904 she studied nursing at Grant Hospital Training School in Columbus, Ohio; and from 1914 to 1915, she studied at Teachers College Public Health Nurse, New York^(20,21)^.

The spelling of her name as “Ethyl” was maintained by our biographer until before her marriage, as it appears in her record in the list of graduates of the 1902 class of the Grant Hospital nursing course^(13)^; however, in the list of graduates of the first graduate class in PH from the Teachers College (1914–1915), her name appears as Ethel Shaman Parsons^(15)^.

On July 20, 1905, already a 23-year-old registered nurse, Ethel Estelle Shaman had her engagement announced in The Journal Herald. In this marriage announcement, her parents were cited as being Mr. and Mrs. James McCann, referring to her stepfather and mother^(13,15)^. On July 15, 1906, at the age of 24, Ethel Estelle Shaman married in Benton Harbor, a city in Berrien County in the U.S. state of Michigan. Her husband, Azariah Worthington Parsons, 49, was a renowned physician and writer. Dr. Parsons, as he was known, had already had two marriages before joining young Ethel: one in 1881 and one in 1885^(13,15,17)^.

While she was married, Ethel lived in Texas with Dr. Parsons. The couple had no children, and divorced after four years of marriage in 1910 when Ethel was 28 years old. In the same year census, she declared herself divorced and the main provider of her family. Dr. Parsons married for the fourth time in 1910, after divorcing Ethel, and died at his home in Oakwood, Texas, on September 4, 1931, of chronic myocarditis^(13,14)^.

### Building a Successful Career: Academic and Professional Life

Ethel took her first job at age 16 as a stenographer at an accounting firm, where she stayed for a year^(13)^. She then became an employee of the American Medical Association in Chicago, from which she resigned on September 14, 1899 to start a job at the Athens Hospital, in the same city, indicated by the Governor of Ohio (administration 1896 to 1900), Asa Smith Bushnell. Ethel, at that time, was single and aged between 17 and 18 years^(15)^.

This activity and profession of her mother probably sparked her interest in nursing, since, in 1902, at the age of 20, she entered the Nursing Training School at Grant Hospital, in Columbus/Ohio, graduating as a nurse two years later^(13,17)^.

Ethel Parsons studied nursing specialization in PH, from 1914 to 1915, at Teachers College – Columbia University, a graduate degree school in education, health and psychology, located in New York. The course was for graduate nurses who, on completion, also received professor certificate. The course was taught by Mary Adelaide Nutting, a Canadian nurse, graduated from the first nursing training program at Johns Hopkins University, in 1891, a model nurse for her participation in important curriculum reforms in the USA, especially the one that originated the Standard Curriculum for Schools of Nursing (1917), proposed by the National League of Nursing^(20,21)^.

Ethel Parsons continued to rise professionally, and, after specializing, began work as a PH nurse in the Houston Settlement, Texas, in 1916. Later that year, she joined the American Red Cross (ARC) nursing services to train and select nurses during the First World War. After the war, Ethel started working as Director of Nursing at the Secretariat of PH in one of the four ARC divisions, where she also trained and registered nurses to work during the Spanish flu epidemic, from February to July 1918. Subsequently, she had to leave work for 19 days due to health reasons, not returning to this coaching role^(19,20)^.

In 1919, after a year of service, Ethel resigned as Director of CVA to work as Nursing Director of the Texas State Secretary, but remained a member of the ARC National Nursing Committee. That same year, she became Director of the Bureau of Child Hygiene, created on September 1, 1919 by the Texas Board of Health, aiming at preventing child deaths and strengthening the health care system, strongly supported by the Director of the Texas Health Board^(16,19)^.

As director, she recommended an active campaign that was more related to child health, due to the country’s general statistics showing that, for every 154 babies born, one mother lost her life and that, in 1918, in the state of Texas alone, 625 mothers died in childbirth. Furthermore, in medical settings, all agreed that 90% of this loss could be prevented and that proper prenatal care and instructions would save mother and baby^(16,19)^.

Concomitantly with her position of director, she was a member of the Health Council for Child Hygiene in Care for Prenatal and Premature Newborns at the State Council of Health and Nursing in PH of America, maintaining her position as District Director of the Red Cross of Texas^(16)^.

In January 1921, she published an article entitled Child Hygiene and Public Health Nursing, in the Texas State Journal of Medicine, in which she briefly described how she organized health services in the Bureau of Child Hygiene, work that allowed her to perfect a cooperative plan between the ARC, the University of Texas, the Child Welfare Division of the General Federation of Women’s Clubs, and the Texas Congress of Mothers. After securing cooperation with these organizations, Ethel Parsons began planning the programs she developed while serving in this state^(17–19)^.

In June 1921, Wickliffe Rose, Director of the RF International Health Board (IHB), sent an invitation letter to Ethel Parsons showing the Brazilian government’s interest in implementing PH nursing services and creating a nursing school in the state of Rio de Janeiro/Brazil^(20,21)^. Ethel Parsons was one of the nurses to present the North American PH program to Brazilian sanitary physician Carlos Chagas, when he went on a trip to the USA with the purpose of learning American nursing^(21)^.

On September 5, 1921, Ethel Parsons arrived in Brazil to coordinate the Technical Cooperation Mission for modern nursing development in Brazil. Its first initiative was to make a situation report, finding that existing nursing schools did not have minimum standards comparable to those adopted in Anglo-Saxon countries, that hospitals were overcrowded and that nursing was made up of people, of both sexes, with no training in nursing^(18,20,21)^.

Inside the DNSP, physicians working in tuberculosis, venereal diseases and child hygiene services had hired 44 girls with a low level of education, who, after twelve lectures, began to act as sanitary visitors. For Ethel Parsons, this situation was inappropriate and compromised nursing care, as visitors lacked basic nursing knowledge concomitantly with systematized training in fields of practice^(20,21)^.

After being hired as a special member of the RF IHB, Ethel Parsons was appointed superintendent of the newly created DNSP Nursing Service and was given the task of supporting the Brazilian government in a health reform, in which she organized the PH nursing service, whose period of operation lasted ten years, starting her work with hygiene visitors in 1921 giving intensive courses of six to ten months on essential subjects of the nursing course while installing a modern nursing school, attached to the General Assistance Hospital, in Rio de Janeiro, then the capital of Brazil^(20)^.

Parsons Mission closed in 1931, when Ethel Parsons, after some requests made to the RF to return to her country, had her request granted. Ethel Parsons requested a return to the USA after spending months investigating a serious health problem that led her to urgent treatment for tuberculosis care in the mountainous region of Rio de Janeiro. The disease was ruled out by physician Thompson Motta after examining her, but there was no diagnosis for what Ethel was feeling at that moment. Ethel also complained of dental problems^(20,21)^.

With the end of the Mission in Brazil, with health problems under investigation, Ethel Parsons returned to Texas on September 15, 1931. The following year, in the city of Las Cruces, New Mexico/USA, Ethel Parsons, then 50 years old, married again, this time to millionaire heir Thomas M. Fairbairn, on June 20, 1932. Since then, there is no news of her work in nursing. Thomas M. Fairbairn died in 1943, aged 69, and now Ethel Parsons was a widow^(20,21)^.

Ethel Estelle Shaman Parsons Fairbairn’s death certificate records her passing on November 3, 1953, at 4:30 am, at St. Benedictos Hospital, Bexas, Texas, due to hypertensive cardiovascular disease, aged 71. She was cremated on November 7, 1953, at Removal Burial Cremation, and her ashes were taken to the Mountain View Mausoleum Cemetery, in Altadena, Los Angeles County, California, where she rests next to her last husband^(13,14)^.

## DISCUSSION

The first point of discussion in Ethel Parsons’ biography concerns the different spellings of her first name and her father’s last name in documents such as registration forms, school lists, among others. Based on studies of American culture in the 19^th^ and early 20^th^ centuries, it is possible to assume that, having been born in a village in Ohio, region of great migration of black and Jewish families, and where English, American (New York), Irish and Russian immigrants settled, it was probably common different pronunciations for her name Ethyle^(27)^.

This would explain the fact that she chose to write Ethel (with “e”), making pronunciation uniform for these different immigrants, then residents of her region, since her name Ethyle must be pronounced as “Étôul”, the same used for Ethel. However, in a mestizo region, it was probably common for people to pronounce “Étíul”, which to the ears of an aristocratic girl could sound uncomfortable. Thus, the correct pronunciation of her name would be more obvious if written “Ethel”, while the spelling with “y”, for “Ethyle” or “Ethyl”, led to sounds and understandings different from the desired one^(13,14)^.

The educational institutions where Ethel studied indicate an excellent school education and show her interest in acquiring knowledge, progressing in her studies to graduate school, attended at an institution that was already important in her time – Teachers College –, where she graduated from the first graduate class in PH, including her name among the former students of 1914–1915^(15,23)^.

It is understood that this religious, social and cultural academic training enabled Ethel to have a differentiated preparation in all fields, including the professional one. This statement is confirmed when assessing her professional experience prior to working in Brazil, and it is possible to observe that she worked in four different activities and places, alternating between leadership, teaching, management, and care activities, focusing her early career years on performances in the South, a region close to the Mexican border, which might explain the information about her speaking Spanish^(20,21)^.

Analyzing these activities, it can be inferred that Ethel Parsons, in addition to being a nurse and educator, was a born leader, with a vision of work management and competence, which was articulated with her patriotic identity as a DAR member^(24)^. These experiences strengthened her qualities to later take on the most important function, according to authors’ view, in her life, which is to coordinate the work of what would be called Parsons Mission in Brazil.

These diverse but complementary experiences enabled Ethel Parsons to gain an understanding of how to meet health needs, both in hospitals and in PH, in times of war and in times of peace. Undoubtedly, she was recognized in the USA as a competent professional in terms of service management, implementation and public health policy development, skilled in relations with institutions, as well as a great leader with functions that she performed concomitantly.

As a scholar, committed to health and nurse role in this process, Ethel Parsons believed that it was everyone’s duty to commit efforts to avoid the unnecessary waste of human lives, not to mention the workforce loss that often occurred. Child health and well-being were essential in any general PH and preventive medicine program, and the prominence that this issue had in Brazil was indicative of its fundamental importance^(19,22)^.

Ethel Parsons’ professional identity was built based on basic education compatible with the profile of women in society at the time, two-year nursing course, specialization at a renowned university, professional practice in the area of specialization and occupation of high administrative positions in health. Ethel was appointed for some of these positions, such as her work at Athens Hospital, by the Governor of Ohio, which was probably due to her family’s prestige and political and social influence^(7,15,16)^.

Thus, it is clear that Ethel Parsons, head of Parsons Mission, played a leading role in female leadership in cooperation project involving the Brazilian government (DNSP) and the North American government (RF), to transform nursing into a recognized profession in Brazil, with its own identity, compatible with modern nursing, differentiating itself from what in the country was carried out as nursing. The Parsons Mission project was successful, as it created a service for PH nurses at federal level and a school for nurses at higher level standards. It should also be noted that, with regard to nursing training, Parsons Mission adopted female exclusivity in the profession, in addition to requirement of specific qualities also contained in the initial model advocated by Florence Nightingale^(25,26)^.

Ethel Parsons’ nursing model considers that, for patients to become the center of nursing care, it is necessary to know their culture, their values and their life expectations, not just offering them what was prescribed by physicians. This added importance to nurses’ home visits, professionals responsible for learning patients’ home environment, their families, their professional role, and their social class, seeking individualized care^(28,29)^.

Ethel Parsons also considered that PH nurses were the most effective agents of the educational campaign instituted in the Health Reform in the USA, which would be promoted along the same lines by Dr. Carlos Chagas in Brazil. She highlighted the importance of a children’s health center, under supervision of a PH nurse, as a place to learn about the best means of preventing diseases among children. She argued that the essential thing in a children’s health center was to have a good physician and a good PH nurse to care for children and mothers and that there was a proper environment for this care^(19,21)^. According to Ethel Parsons, the PH team at the time included, which in Brazil would also be constituted like this, physicians and PH nurses, who were the professional authorities in a health center.

Also in Ethel Parsons’ writings, she stated that PH nurses’ growth occurred in response to a need in the field of medicine. However, to meet the demanding standards of these professionals, good training in a general hospital and at least two months of special training in PH nursing were required. According to Ethel Parsons, nurses, in order to be successful in their work in PH, should be endowed with a true love for humanity and a spirit of work and teaching, which somehow reflects their training in Presbyterian principles^(20,22)^.

The articles by Ethel Parsons bring us characteristics of her social and professional identity, which especially valued PH nurse care, with a high standard of knowledge, capable of mobilizing communities in favor of health prevention, starting with maternal and child health. Similarly, her social identity as an aristocratic Christian woman, who inherited her grandfather’s patriotic legacy, is clear when she associates humanitarian values and an attitude of serving the profession with PH nurse characteristics^(22)^.

The work she developed, articulating health education, preparing professionals for PH nursing work and quality training, in addition to the texts she published on how health should be promoted and developed in the community, converged with RF’s ideals in terms of providing health education possibilities for other developing American countries, especially Brazil, establishing friendly political relations and increasing USA cultural influence on Latin American countries^(28–30)^.

The Parsons Mission produced, in 10 years in the country, extraordinary effects, such as implementation and recognition of a high-standard nursing school, organization of a national service of PH nurses, rearrangement of nursing in several federal hospitals, including the General Care Hospital, creation of an association of nurses, which won a seat on the International Council of Nursing (ICN) in 1929, and enactment of legislation that regulated nursing practice in Brazil. Moreover, giving visibility to the figure of Registered Nurses reinforced the image of an economically emancipated woman in the collective imagination, which can be considered bold for the period^(29,30)^.

Thus, Ethel Parsons’ biography reaches a degree of relevance not restricted only to her initial mission in Brazil, but to its extension as a model of nursing education that has influenced all periods until then. Without a doubt, her life story must be known and celebrated, as she carves out an identity as a nurse that projects itself into the 21^st^ century in all the struggles of nursing.

This study does not exhaust the knowledge that can be produced about Ethel Parsons’ personality, as it was partially limited by the difficulty in accessing written documents, since most of them are disseminated in institutions of different North American states, and acquiring them required a large investment of research time with requests and negotiations by email and phone call, in addition to paying fees and hiring an English translator.

## CONCLUSIONS

This biographical research sought to explore the processes of genesis and development of Ethel Parsons’ personality in the social spaces in which she was inserted and how they shaped her experiences in nursing. Being able to reveal facts never before accessed in her biography makes nursing history researchers give new meaning to situations and events surrounding their existence.

Ethel Parsons can be considered one of the forerunners of the struggle for nursing care by formally trained personnel. She was an example of profile of PH nurses not yet known in Brazil, without, however, giving due value to the training of hospital nurses, facts that demonstrate a construction of a comprehensive professional identity in line with advances in nursing.

Her work had great repercussions in all Brazilian states and her name is among the pioneers of nursing in the country, due to the great health campaign she initiated, raising the health status of a resource-poor population, and having provided young women in the country with a new field of action, profession, financial independence and social recognition, by allowing them to enter the nursing profession, which came to be well regarded by Brazilian society at the time.

Knowing Ethel Parsons’ biography characteristics allows us to broaden the understanding of who we are and how much we can become in the world nursing scenario. One hundred years after her arrival in Brazil, Ethel Parsons’ biography today represents a vision of the future and vanguard for all who are starting in nursing.
